# A Comparison of Simple Methods to Incorporate Material Temperature Dependency in the Green’s Function Method for Estimating Transient Thermal Stresses in Thick-Walled Power Plant Components

**DOI:** 10.3390/ma9010026

**Published:** 2016-01-06

**Authors:** James Rouse, Christopher Hyde

**Affiliations:** Department of Mechanical, Materials and Manufacturing Engineering, University of Nottingham, Nottingham, Nottinghamshire NG7 2RD, UK; Christopher.Hyde@nottingham.ac.uk

**Keywords:** thermal fatigue, header, Green’s function method, power plant, P91

## Abstract

The threat of thermal fatigue is an increasing concern for thermal power plant operators due to the increasing tendency to adopt “two-shifting” operating procedures. Thermal plants are likely to remain part of the energy portfolio for the foreseeable future and are under societal pressures to generate in a highly flexible and efficient manner. The Green’s function method offers a flexible approach to determine reference elastic solutions for transient thermal stress problems. In order to simplify integration, it is often assumed that Green’s functions (derived from finite element unit temperature step solutions) are temperature independent (this is not the case due to the temperature dependency of material parameters). The present work offers a simple method to approximate a material’s temperature dependency using multiple reference unit solutions and an interpolation procedure. Thermal stress histories are predicted and compared for realistic temperature cycles using distinct techniques. The proposed interpolation method generally performs as well as (if not better) than the optimum single Green’s function or the previously-suggested weighting function technique (particularly for large temperature increments). Coefficients of determination are typically above 0.96, and peak stress differences between true and predicted datasets are always less than 10 MPa.

## 1. Introduction

There is a clear need in many industries to be able to predict the long-term behaviour of components operating in demanding environments in order to prevent/understand material failure. The aim is that with a greater understanding of how a component reacts due to a particular loading pattern, remnant life can be quantified. With this confidence, plant efficiency and longevity could be maximised safely. In particular, pressure is mounting on thermal power plant operators to generate electricity in an efficient and economical manner. Unit loads are expected to fluctuate with higher frequencies and steeper “ramp up and down” rates as drivers attempt to match market demands. Such so-called “two-shifting” or “partial-load” operating conditions have been in use for many years [1]; however, concern over their implementation is mounting as the amount of time a plant has to come on line reduces [2]. Generally, as steam pressures and temperatures vary with time, potentially large thermal stresses will develop in thick-walled components, such as steam headers. The fluctuation of total stress (mechanical and thermal) in components makes fatigue an important structural integrity concern in power plant components; a problem that is significantly complicated by the transient nature of thermal stresses. The present work looks to establish a technique based on the Green’s function method that estimates transient thermal stresses while accounting for temperature-dependent material properties.

Many novel monitoring systems have been developed for assessing the structural integrity of at-risk power plant components, including “on line” management systems that monitor power station load characteristics (such as main steam temperature and pressure) and estimate component degradation using generalised finite element models and creep/fatigue damage fraction rules [3,4,5,6]. An example of one of these products is Areva’s fatigue monitoring system FAMOSi (Erlangen, Germany) [7,8], where thermal loads are recorded using on site thermocouples and converted to thermal stresses using FEA (finite element analysis) models at critical points in a system. Alternatively, accurate stress histories in a component may be estimated through bespoke analyses utilising complex visco-plastic material models [9]; however, this is commonly computationally intensive and is typically impractical for on line component assessment.

While these advances have shown some success, established design codes and analysis procedures are still by far the most commonly-used tools in industry for component fitness assessment, along with frequent inspection during outage periods [10]. In the UK, the R5 [11,12] procedure is commonly used for high temperature assessment and the R6 [13] procedure for low temperature fracture assessment of power plant components. These step by step methods usually involve decomposing a loading history into cycles. The likelihood of failure by various mechanisms, such as plastic collapse, creep and fatigue, is calculated by estimating damage accumulation and mechanism interaction factors.

The Green’s function method provides a general approach to estimate the transient linear elastic thermal stress responses at a point in a structure by integrating the response due to a unit thermal load change. In the context of steam headers, thermal stress histories may be estimated at a point of interest for any bulk steam temperature history. While limited to linear analysis (due to the inherent summation during integration), the Green’s function method is still of use in component failure assessment, particularly where damage is suspected to be localised. The Green’s function method (see Section 2.2) has been show to be a useful tool in predicting transient thermal stresses by several authors. In particular, the technique has been applied to fatigue analysis problems in the nuclear power industry [14,15,16]. It has often been assumed (for simplicity of integration) that Green’s functions are temperature independent. In reality, this is not the case due to variations in material properties with temperature. The work of Koo *et al*. suggested the implementation of a temperature-dependent weighting function [17]; however, this neglects second order variations (*i.e.*, it assumes time-independent scaling of Green’s function). The present work looks to compare this method to a developed interpolation procedure in order to establish the importance of these second order effects.

## 2. Background

### 2.1. The Thermoelastic Problem

The governing equations for a linear coupled thermoelastic problem may be derived from the fundamental principles of mechanics and thermodynamics. When loads applied to a body give rise to variations in strain within the body, variations in temperature are also observed. This causes heat flow and therefore an increase in entropy for the body (this irrecoverable mechanical dissipation is known as thermoelastic dissipation). There is an internal generation of heat due to mechanical deformation that will affect the temperature field within a body in addition to any thermal boundary conditions. Deformation, however, is not only controlled by the application of, say, body forces. Temperature fields cause thermal expansion within elements of the body, generating additional internal surface forces between the elements. There exists therefore a coupling between the solutions for temperature and displacement fields, ***T***(P,t) and ***u***(P,t), respectively (where *P* is a point within the body specified by coordinates using the coordinate system $x_{1}$, $x_{2}$, $x_{3}$ = *x* and *t* is time). For a linear coupled thermoelastic problem, it may be shown that a unique solution may be found (for a given set of initial and boundary conditions) using the heat equation with mechanical coupling (Equation (1)), the equilibrium condition (Equation (2); note the inclusion of an inertia term on the right hand side of the equation), the strain-displacement relations (Equation (3)) and the stress-strain relations (Equation (4)) [18]. Note the use of indicial notation ($\frac{\delta g_{i}}{\delta x_{j}}=g_{i,j}$, where $g_{i}$ is a vector component in the *i*-th direction and $x_{j}$ is the basis vector in the *j*-th direction of the coordinate system, i,j = 1,2,3) and the Einstein summation convention. Note also that dots are used to denote derivatives with respect to time.

(1) $$kT_{,mm}=\rho C\dot{T}+\left(3\lambda +2\mu \right)\alpha T_{0}{\dot{\epsilon }}_{kk}$$

(2) $$\sigma_{ij,j}+f_{i}=\rho {\ddot{u}}_{i}$$

(3) $$\epsilon_{ij}=\frac{1}{2}\left(u_{i,j}+u_{j,i}\right)$$

(4) $$\sigma_{ij}=\delta_{ij}\lambda \epsilon_{kk}+2\mu \epsilon_{ij}-\delta_{ij}\left(3\lambda +2\mu \right)\alpha T$$

where *ϵ*, *σ* and *f* are the small strain tensor, the stress tensor and the body force vector field, respectively; $T_{0}$ is a reference temperature at which, in the absence of body forces, the material will be in a stress-free state. The material-dependent parameters are thermal conductivity (*k*), density (*ρ*), specific heat capacity at constant deformation (*C*) and the thermal expansion coefficient (*α*). Lamé’s first and second parameter are defined in terms of Young’s modulus (*E*) and Poisson’s ratio (*ν*) in Equation (5). $\delta_{ij}$ is the Kronecker delta ($\delta_{ij}=1$ if i=j, else $\delta_{ij}=0$).

(5) $$\begin{array}{cc} \lambda & =\frac{E\nu }{\left(1+\nu \right)\left(1-2\nu \right)}\\ \mu & =\frac{E}{2\left(1+\nu \right)} \end{array}$$

The existence of the coupling term in the energy equation (Equation (1)) greatly complicates the solution process for the thermoelastic problem (clearly, temperature and displacement field solutions must be found simultaneously to satisfy Equations (1)–(4) and the problem-specific initial and boundary conditions). In general, temperature variations due to mechanical deformations are small (particularly if the small strain theory is implemented). Similarly, differences between heat transfer solutions in deformed and undeformed bodies are also small (deformations from either thermal expansion or external mechanical agencies do not change the dimensions of the structure to such an extent that heat transfer is significantly affected). If thermoelastic dissipation can be neglected (as is almost always the case [18]), an uncoupled formulation may be derived for the thermoelastic problem. In this case, internal heat generation due to deformation is ignored, and temperature fields can be found first by solving the well-known heat equation (Equation (6)), where *κ* is the thermal diffusivity (κ=k/ρC). Once the temperature field has been determined, the corresponding displacement field (dependent on thermal expansion and mechanical body forces) may be found.

If the rate of change of the deformation rates are small (as is the case in many engineering applications), inertia effects may be neglected, and the formulation is termed quasi-static. In this case (with the absence of body forces), the equilibrium equation simplifies to Equation (7). Strain-displacement and stress-strain relations given in Equations (3) and (4), respectively, are still valid in the uncoupled formulation.

(6) $$\kappa T_{,mm}=\dot{T}$$

(7) $$\sigma_{ij,j}=0$$

### 2.2. The Green’s Function Method for Predicting Transient Thermal Stresses

The thermoelastic problem has been defined in Section 2.1. Solutions for even the uncoupled formulation with the component geometry used in the present work (power plant steam header) are very complex and generally require numerical methods (such as finite element analysis (FEA)) to estimate a solution. A practical problem with this analysis strategy is that temperature, displacement and, consequently, stress fields would need to be found for each new operating condition. It is not feasible to perform full FEA simulations of the header components for each change to the bulk steam temperature or pressure. A solution to this dilemma however exists through the use of Green’s functions.

It can be seen from Section 2.1 that the thermal stress solution is based on the temperature field solution, both of which are unique and dependent on the particular initial and boundary conditions of the problem. This uniqueness allows the use of a Green’s function that finds the thermal stresses based on the boundary conditions. It is therefore possible to determine the thermal stress distribution without direct knowledge of the temperature or displacement fields. As the present work is concerned with power plant header applications, the bulk internal steam temperature may be used as a “driving” term for the thermal stress field (it shall be assumed that external surfaces of the header are insulated, and attention is restricted to the uncoupled formulation). Thermal stresses at a point *P* in the header structure can be found by the integral in Equation (8), where G(P,t−τ) is Green’s function, ψ(t) is the bulk internal steam temperature and *τ* is the time integration variable.

(8) $$\sigma \left(P,t\right)=\int_{0}^{t}G\left(P,t-\tau \right)\frac{d\psi (\tau )}{d\tau }d\tau$$

Numerical integration of Equation (8) may be accomplished using Equation (9), where $G_{SS}\left(P\right)=\lim_{t\to \inf}G\left(P,t\right)$. In the present work, the temperature field is allowed to reach equilibrium, and mechanical loads are not considered; therefore, $G_{SS}\left(P\right)=0$.

(9) $$\sigma \left(P,t\right)=G_{SS}\left(P\right)\psi \left(\tau \right)+\sum_{t-t_{CH}}^{t}\bar{G}\left(P,t-\tau \right)\Delta \psi \left(\tau \right)$$

$\bar{G}\left(P,t-\tau \right)$ represents the thermal stress response due to a unit temperature step at the point of interest *P* (assuming no other loads are present in the structure). Green’s function may be represented by a sum of exponential terms (see Equation (10)).

(10) $$\bar{G}\left(P,t\right)=\exp\left(\sum_{m=1}^{7}C_{m}\left(P\right){\left(\ln(t)\right)}^{m-1}\right)$$

The work of Koo *et al.* introduced temperature dependency in the Green’s function method using a weighting function dependent on the bulk temperature ($W\left(\psi \right)$) [17]; see Equation (11). Note, for simplicity, this method will be refereed to as the “weight function” method for the reminder of the present work. Based on the work of Koo *et al*., a fourth order polynomial has been assumed for use in a comparison study (see Equation (12)).

(11) $$\sigma \left(P,t\right)=\int_{0}^{t}G\left(P,t-\tau \right)W\left(\psi \right)\frac{d\psi (\tau )}{d\tau }d\tau$$

(12) $$W\left(\psi \right)=A_{0}+A_{1}\psi +A_{2}\psi^{2}+A_{3}\psi^{3}+A_{4}\psi^{4}$$

## 3. FEA Header Models and Temperature-Dependent Material Properties

**Figure 1:**
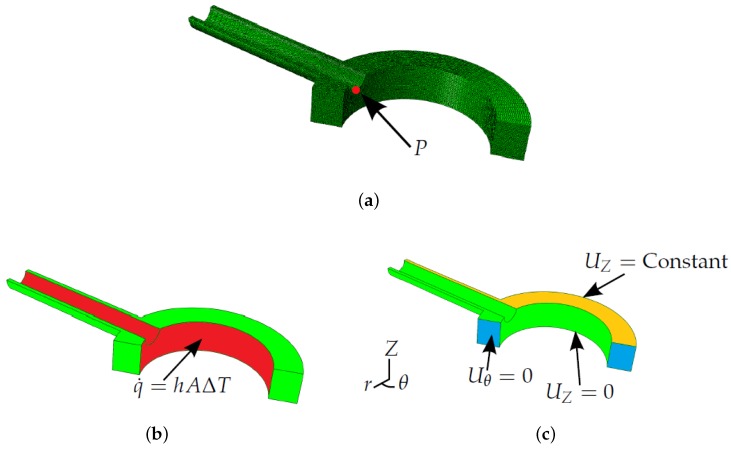
Finite element analysis (FEA) models, showing: (**a**) the tetrahedral mesh, exploiting the plane of symmetry between stub penetrations and showing the location of the example point of interest *P*; (**b**) boundary conditions in the thermal analyses; and (**c**) boundary conditions in the mechanical analyses.

FEA models must be generated in order to determine the coefficients in Equation (10) and, thus, to define Green’s functions. FEA has been conducted in the present work using the commercially-available code ABAQUS (Dassault Systèmes, Paris, France). Since the present work looks to establish a method to introduce temperature dependency in the Green’s function method, a simplified two-stub penetration model has been used by way of example (see Figure 1a, noting the plane of symmetry assumed between stub penetrations). Despite the simplified geometry, shell and stub dimensions are similar to those found in industry for P91 header components. Uncoupled thermoelastic analysis was conducted by first determining a temperature field from a heat transfer simulation. An insulated exterior boundary condition was assumed ($\dot{q}=0$) to allow temperature fields in the model to reach equilibrium after the bulk steam temperature experiences a step change. Heat conduction on the inside surface of the header is controlled by convection (see Figure 1b), where the heat transfer coefficient *h* is taken to be a temperature-independent constant 0.002 W/mm${}^{2}$K. Once the transient temperature field has been determined, it can be used as an input in mechanical analyses to estimate thermal stress histories. Boundary conditions for the mechanical analysis can be seen in Figure 1c. An “equation” type constraint [19] is applied to the upper surface of the model (designated by the label $U_{Z}=\text{Constant}$ in Figure 1c). This enforces equal displacements in the *Z* direction between all nodes on this plane, thus ensuring it remains planar, and the assumed symmetry holds. Tetrahedral quadratic elements where used, namely DC3D10 (ABAQUS) for thermal analyses and C3D10 (ABAQUS) for mechanical analyses (see Figure 1a for an example mesh) [19]. In has been indicated in the literature that ligament cracking is a potential concern for header components, notably when thermal fatigue is a significant damage mechanism [20]. As discussed previously, the Green’s function method allows for estimation of stresses at a singular analysis point only. With these factors in mind and for the illustration of the stress analysis potential of the Green’s function method, an analysis point (*P*; see Figure 1a) is considered in the present work that represents crack initiation at the inner bore [20].

**Figure 2:**
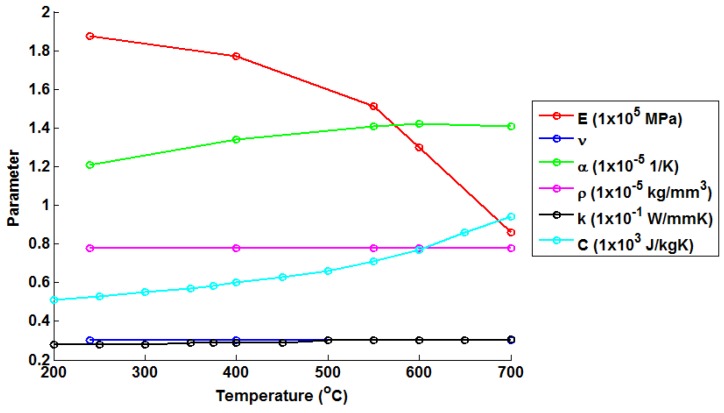
A summary of the temperature-dependent material properties used in the present work to represent a P91 chrome steel.

A single material is assumed for the FEA model in the present work (variations in material properties at the stub weld are not considered). Temperature-dependent material parameters are required in order to calculate transient thermal stresses within the header models. Values for Young’s modulus (*E*) and the instantaneous thermal expansion coefficient (*α*) have been determined from monotonic tests performed on an Instron 8862 thermomechanical fatigue machine (operating under isothermal conditions, Norwood, MA, USA) utilising radio frequency induction heating and using a TA instruments Q400 thermomechanical analyser (New Castle, DE, USA), respectively (see Table 1). Tested temperature ranges were chosen to represent the typical bounds of operation for thermal power plant components. The remainder of the material constants have been taken from the work of Yaghi *et al*. [21] (Table 2). A negligible dependency is assumed in density (*ρ*) and Poisson’s ratio (*ν*) over the tested temperature range. As such, values for these quantities are taken to be 7.76 × $10^{-6}$ kg/mm${}^{3}$ and 0.3, respectively. Temperature dependent material properties are summarised in Figure 2.

**Table 1:** A summary of the temperature-dependent material parameters (representative of a P91 chrome steel), determined through experimental analysis, used in the FEA modelling.

Temperature (℃)	Young’s Modulus (*E*) (N/mm${}^{2}$)	Thermal Expansion Coefficient (*α*) (1/K)
240	1.88 × $10^{5}$	1.21 × $10^{-5}$
400	1.77 × $10^{5}$	1.34 × $10^{-5}$
550	1.51 × $10^{5}$	1.41 × $10^{-5}$
600	1.30 × $10^{5}$	1.42 × $10^{-5}$
700	8.58 × $10^{4}$	1.41 × $10^{-5}$

**Table 2:** A summary of the temperature-dependent material constants (representative of a P91 chrome steel), taken from the work of Yaghi *et al*. [21], used in the FEA modelling.

Temperature (℃)	Thermal Conductivity (*k*) (W/mm.K)	Specific Heat Capacity (*C*) (J/kg.K)
200	0.028	510
250	0.028	530
300	0.028	550
350	0.029	570
375	0.029	585
400	0.029	600
450	0.029	630
500	0.03	660
550	0.03	710
600	0.03	770
650	0.03	860
700	0.0305	942

**Figure 3:**
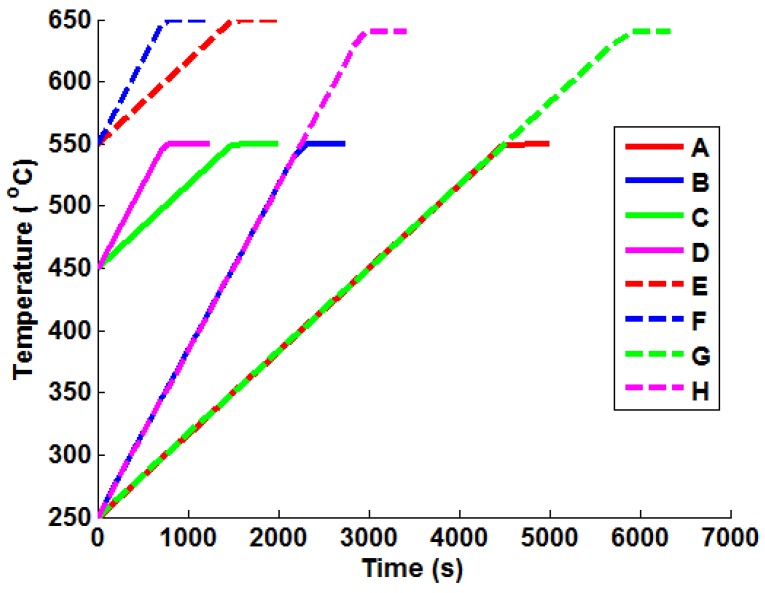
Plots of the representative ramp up temperature profiles used to test the various Green’s function implementation techniques.

**Figure 4:**
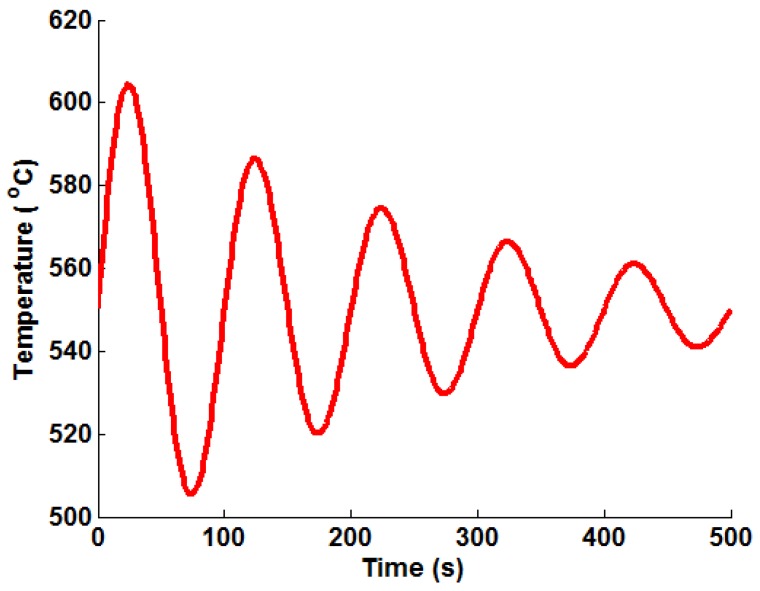
A plot of the representative oscillating (decay) temperature profile used to test the various Green’s function implementation techniques.

In order to apply the Green’s function method and the temperature interpolation techniques, realistic thermal stress histories must be generated from the FEA models for representative bulk steam temperature profiles (mechanical loading is neglected here, as it is a trivial exercise to scale linear elastic loads based on varying internal pressures). Several “ramp up” temperature profiles have been generated and analysed using the uncoupled thermoelastic procedure (with temperature-dependent material parameters). These are summarised in Table 3 and are representative of the high end limiting bulk steam temperature increments and rates seen in two shifting plant. Plots of the ramp up temperature profiles can be seen in Figure 3 with their respective labels (which are used in the remainder of the present work). An oscillating temperature profile (that represents a control signal correcting bulk steam temperature to a nominal operating temperature of 550 ℃) is also considered (see Figure 4), designated Profile “I”. This profile was generated using Equation (13), using the parameters $B_{1}=60$, $B_{2}=-3.978$ × $10^{-3}$, $B_{3}=\pi /50$ and $T_{M}=550$ ℃.

(13) $$T\left(t\right)=B_{1}e^{B_{2}t}sin\left(B_{3}t\right)+T_{M}$$

**Table 3:** A summary of bulk steam temperature “ramp up” profiles.

Load Case	Start Temperature (℃)	End Temperature (℃)	Temperature Rate (℃/min)
A	250	550	4
B	250	550	8
C	450	550	4
D	450	550	8
E	550	650	4
F	550	650	8
G	250	640	4
H	250	640	8

## 4. Methodology Overview

**Figure 5:**
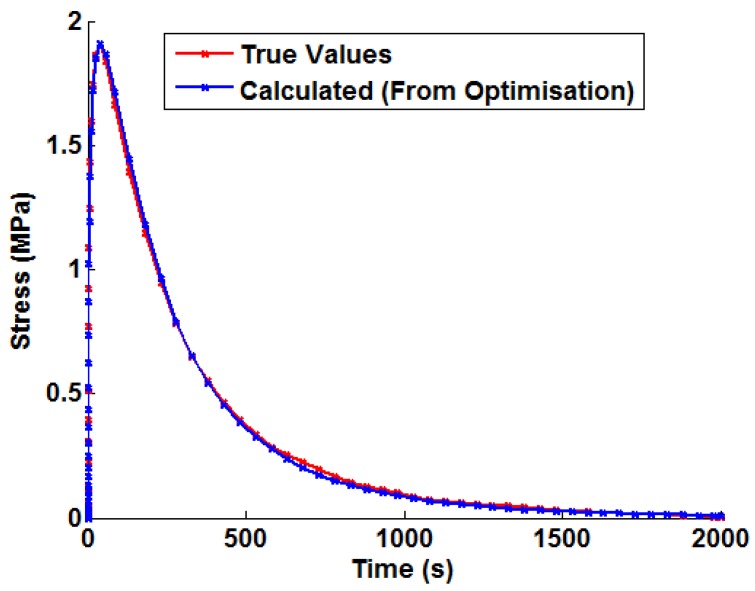
An example of the Green’s function approximation (shown in Equation (10)) fitted to the von Mises stress history from an uncoupled thermoelastic FEA simulation (unit temperature step).

Prior to discussing the proposed method to introduce temperature dependency, it is worthwhile briefly discussing the procedure to determine temperature-independent Green’s functions. Once the thermally-driven stress profile has been determined from FEA for a unit bulk steam temperature step, the Green’s function approximation shown in Equation (10) can by fitted (an example may be seen in Figure 5). This defines the constants $C_{m}$, m=1...7. A non-linear least squares optimisation algorithm (the Levenberg—Marquardt algorithm) was used in a MATLAB program (function LSQNONLIN [22], MathWorks, Natick, MA, USA) to optimise the values of Equation (10) in order to fit the FEA solution.

**Figure 6:**
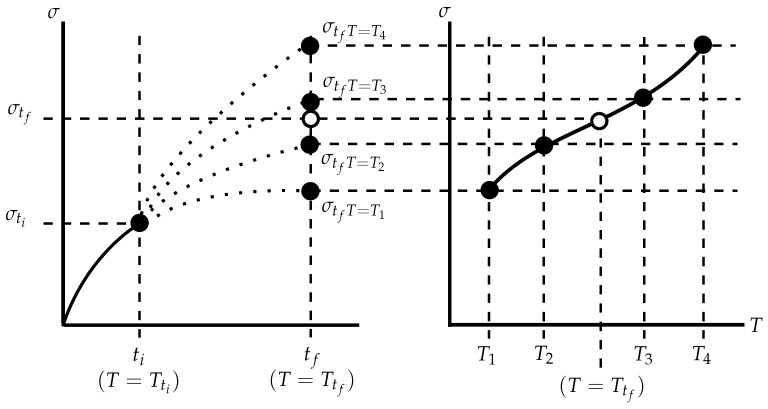
A schematic of the proposed interpolation technique.

A schematic of the proposed interpolation technique is given in Figure 6. The Green’s function method fundamentally relies on determining a reference solution for a unit temperature step that may be integrated for a particular thermal loading history. By including temperature dependency, this reference solution will need to be altered over the thermal history. The proposed technique achieves this by interpolating between solutions determined from temperature-independent Green’s functions. Green’s functions are determined for unit temperature steps, each of which has an associated representative temperature (taken here to be the mean temperature for the unit step). Given some initial conditions (*σ* = $\sigma_{t_{i}}$, *T* = $T_{t_{i}}$), stress increments can be determined for each Green’s function ($\sigma_{t_{f}T=T_{1}}$, $\sigma_{t_{f}T=T_{2}}...$, and so on, with the representative temperatures $T_{1},T_{2},...$; see Equation (14)). Note that a different (temperature dependent) set of constants ($C_{m},m=1...7$) is used to find each reference solution ($\sigma_{t_{f}T=T_{1}},\sigma_{t_{f}T=T_{2}}...$). These constant sets are designated $C_{mT=T_{1}},C_{mT=T_{2}},...$ for representative temperatures $T_{1},T_{2},...$, respectively, in Equation (14). The relationship between the representative temperatures and reference stress values may then be used to interpolate to the actual instantaneous temperature $T_{t_{f}}$ and, thus, find the estimated stress increment $\sigma_{t_{f}}$. A suitably high order polynomial may be used to model this relationship. For *M* reference solutions and representative temperatures, Equation (15) may be used, where $D_{i},i=1...M$ are the coefficients of the polynomial. Generating the reference curves in this way allows the relationship between stress values predicted by particular Green’s functions at a time instant to change with time, thus allowing the second order effect ($\frac{\delta^{2}\sigma }{\delta t\delta T}$) to be accounted for.

(14) $$\begin{array}{cc} \sigma_{t_{f}T=T_{1}} & ={\bar{G}}_{T=T_{1}}\left(P,t_{f}\right)=\exp\left(\sum_{m=1}^{7}C_{mT=T_{1}}\left(P\right){\left(\ln(t_{f})\right)}^{m-1}\right)\\ \sigma_{t_{f}T=T_{2}} & ={\bar{G}}_{T=T_{2}}\left(P,t_{f}\right)=\exp\left(\sum_{m=1}^{7}C_{mT=T_{2}}\left(P\right){\left(\ln(t_{f})\right)}^{m-1}\right)\\ \sigma_{t_{f}T=T_{3}} & ={\bar{G}}_{T=T_{3}}\left(P,t_{f}\right)=\exp\left(\sum_{m=1}^{7}C_{mT=T_{3}}\left(P\right){\left(\ln(t_{f})\right)}^{m-1}\right)\\ & ... \end{array}$$

(15) $$\sigma_{t_{f}}\left(T\right)=\sum_{i=1}^{M}D_{i}T^{i-1}$$

## 5. Results

### 5.1. Unit Temperature Steps

**Figure 7:**
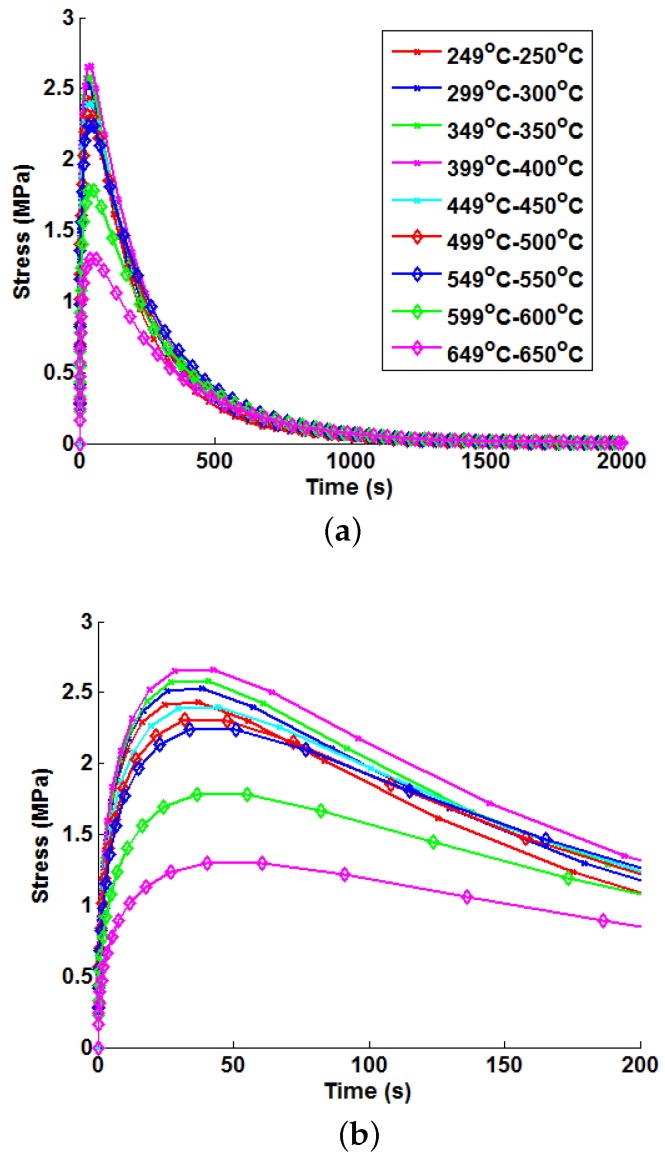
Variations in thermal stress responses due to unit step bulk steam temperature increments, showing: (**a**) general development and decay behaviour; and (**b**) magnified thermal stress development behaviour.

Thermal stress profiles were generated at the analysis point (*P*) defined in Figure 1a for unit bulk steam temperature changes using the modelling techniques discussed in Section 3. Figure 7 highlights the difference in the thermal stress responses due to a unit temperature step (as a result of temperature-dependent material properties). For the analysis point considered, the hoop stress is the dominant principal stress, and the other principal stresses are negligible. Equivalent von Mises stresses are presented here, but these (as far as the Green’s functions are concerned) may be taken to be the absolute of the maximum principal stress.

Similar general trends are observed for all six components of the symmetric stress tensor (there is development of thermal stress until a maximum value is achieved, after which the thermal stress exponentially decays). Equation (10) may therefore be used in general to describe the unit thermal response of for the six stress components at a specific analysis point. Transient thermal stress behaviour may then be approximated as before using the integration shown in Equation (9). Additionally, the temperature dependency method described in Section 4 may also be implemented for each of the stress components. The present work only considers the von Mises stress. This provides a concise and easy to follow way to compare the applicability of the tested methods. If a user were to determine Green’s functions for all six stress components, the von Mises Green’s function could be generated with little additional effort and be used as a constraint to limit any interpolation errors accumulated when processing these individual components.

Peak thermal stress values are observed for the 399 ℃–400 ℃ case. Referring to Figure 2, it can be seen that this is due to the significant increase in the thermal expansion coefficient *α* and the specific heat capacity *C*. This results in higher internal forces being required for a particular temperature gradient (note that thermal conductivity *k* is relatively stable over the given temperature range). After 400 ℃, a marked reduction in Young’s modulus (*E*) is observed, leading to a loss of stiffness in the material and, hence, a reduction in peak thermal stresses. Green’s functions (defined by Equation (10)) have been fitted to each unit step using the method discussed in Equation (4). In all cases, the optimisation terminated due to the change in the sum of squares between iterations falling below a tolerance (set to 1 × $10^{-7}$), suggesting convergence on a local minimum. A summary of the Green’s function coefficients can be seen in Table 4. The weighting function described by Equations (11) and (12) has also been fitted to the unit responses using the same optimisation technique and satisfying the same stopping criterion. A summary of these coefficients may be found in Table 5.

**Table 4:** A summary of Green’s function coefficients fitted to the unit thermal stress responses.

Unit Step	$C_{1}$	$C_{2}$	$C_{3}$	$C_{4}$	$C_{5}$	$C_{6}$	$C_{7}$
249 ℃–250 ℃	−7.07 × $10^{-2}$	4.12 × $10^{-1}$	−2.44 × $10^{-2}$	−2.77 × $10^{-4}$	−1.00 × $10^{-4}$	−3.16 × $10^{-4}$	4.07 × $10^{-6}$
299 ℃–300 ℃	−4.78 × $10^{-2}$	4.14 × $10^{-1}$	−2.40 × $10^{-2}$	−2.99 × $10^{-4}$	−1.00 × $10^{-4}$	−2.97 × $10^{-4}$	2.30 × $10^{-6}$
349 ℃–350 ℃	−4.19 × $10^{-2}$	4.17 × $10^{-1}$	−2.37 × $10^{-2}$	−3.30 × $10^{-4}$	−1.00 × $10^{-4}$	−2.97 × $10^{-4}$	2.56 × $10^{-6}$
399 ℃–400 ℃	−3.16 × $10^{-2}$	4.20 × $10^{-1}$	−2.32 × $10^{-2}$	−3.86 × $10^{-4}$	−1.00 × $10^{-4}$	−2.70 × $10^{-4}$	3.89 × $10^{-8}$
449 ℃–450 ℃	−1.58 × $10^{-1}$	4.21 × $10^{-1}$	−2.29 × $10^{-2}$	−3.65 × $10^{-4}$	−1.00 × $10^{-4}$	−2.52 × $10^{-4}$	−1.24 × $10^{-6}$
499 ℃–500 ℃	−2.16 × $10^{-1}$	4.25 × $10^{-1}$	−2.24 × $10^{-2}$	−4.11 × $10^{-4}$	−1.00 × $10^{-4}$	−2.48 × $10^{-4}$	−1.23 × $10^{-6}$
549 ℃–550 ℃	−2.75 × $10^{-1}$	4.28 × $10^{-1}$	−2.18 × $10^{-2}$	−4.47 × $10^{-4}$	−1.00 × $10^{-4}$	−2.17 × $10^{-4}$	−4.01 × $10^{-6}$
599 ℃–600 ℃	−5.38 × $10^{-1}$	4.31 × $10^{-1}$	−2.13 × $10^{-2}$	−3.35 × $10^{-4}$	−1.00 × $10^{-4}$	−1.97 × $10^{-4}$	−5.30 × $10^{-6}$
649 ℃–650 ℃	−9.02 × $10^{-1}$	4.35 × $10^{-1}$	−2.11 × $10^{-2}$	−1.56 × $10^{-4}$	−1.00 × $10^{-4}$	−1.69 × $10^{-4}$	−7.19 × $10^{-6}$

**Table 5:** Coefficient values determined for the weighting function approach.

$C_{1}$	−8.04 × $10^{-1}$
$C_{2}$	5.13 × $10^{-1}$
$C_{3}$	−2.16 × $10^{-2}$
$C_{4}$	−7.68 × $10^{-3}$
$C_{5}$	−7.19 × $10^{-6}$
$C_{6}$	6.08 × $10^{-5}$
$C_{7}$	−3.17 × $10^{-5}$
$A_{0}$	1.00 × $10^{-1}$
$A_{1}$	−1.43 × $10^{-2}$
$A_{2}$	4.64 × $10^{-5}$
$A_{3}$	−7.71 × $10^{-8}$
$A_{4}$	5.45 × $10^{-11}$

### 5.2. Representative Temperature Profiles

**Figure 8:**
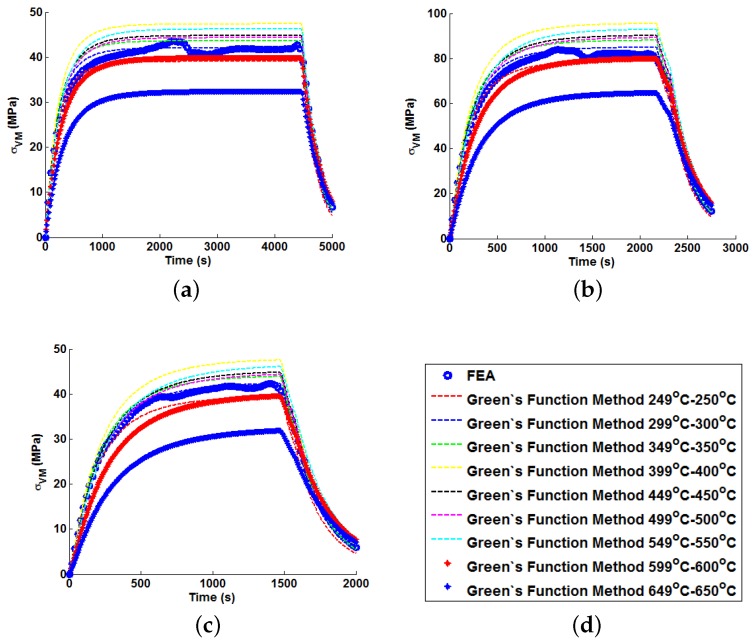
Predictions of thermal stress responses determined using individual Green’s functions for: (**a**) ramp up Profile A; (**b**) ramp up Profile B; and (**c**) ramp up Profile C. Sub figure (**d**) shows the legend used in the plots.

**Figure 9:**
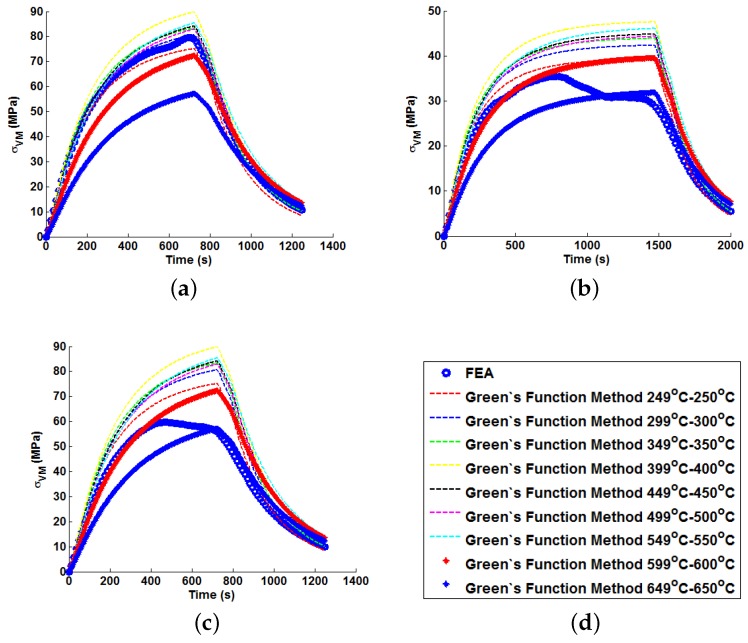
Predictions of thermal stress responses determined using individual Green’s functions for: (**a**) ramp up Profile D; (**b**) ramp up Profile E; and (**c**) ramp up Profile F. Sub figure (**d**) shows the legend used in the plots.

**Figure 10:**
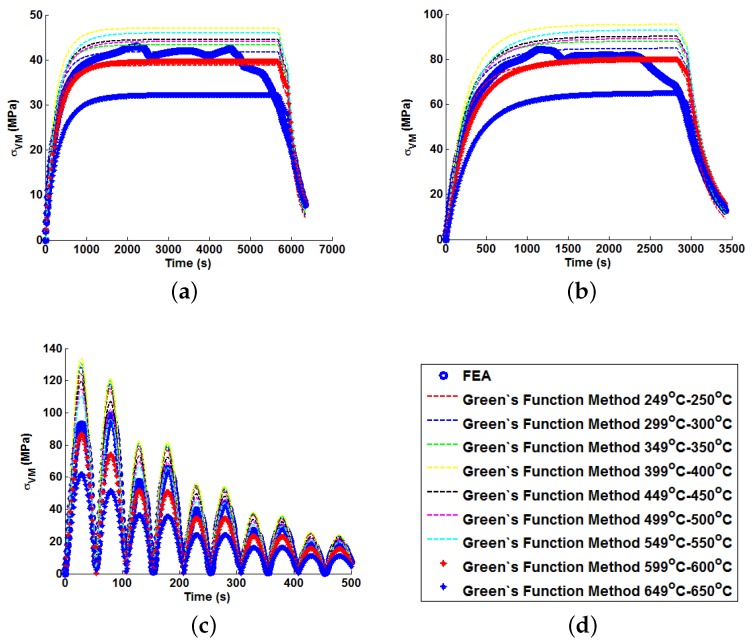
Predictions of thermal stress responses determined using individual Green’s functions for: (**a**) ramp up Profile G; (**b**) ramp up Profile H; and (**c**) oscillating Profile I. Sub figure (**d**) shows the legend used in the plots.

**Figure 11:**
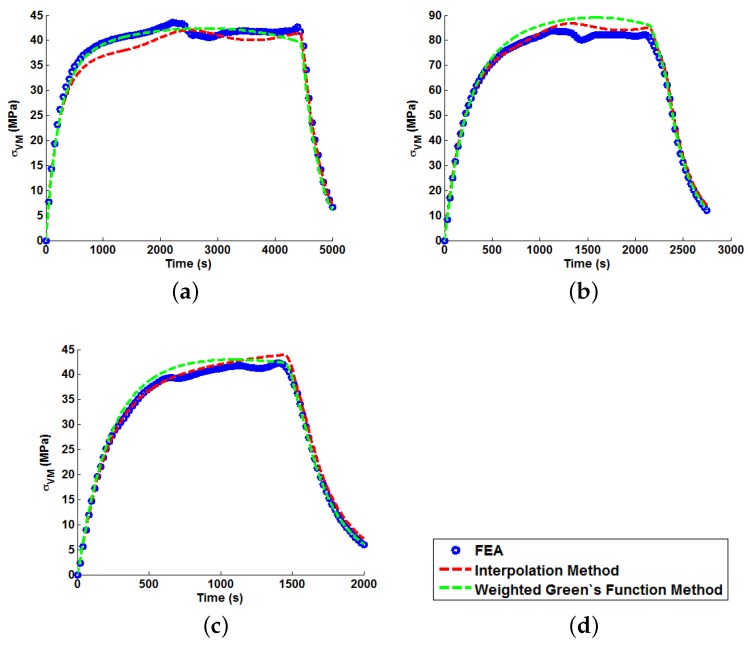
Predictions of thermal stress responses determined using the interpolation and weighted Green’s functions methods for: (**a**) ramp up Profile A; (**b**) ramp up Profile B; and (**c**) ramp up Profile C. Sub figure (**d**) shows the legend used in the plots.

**Figure 12:**
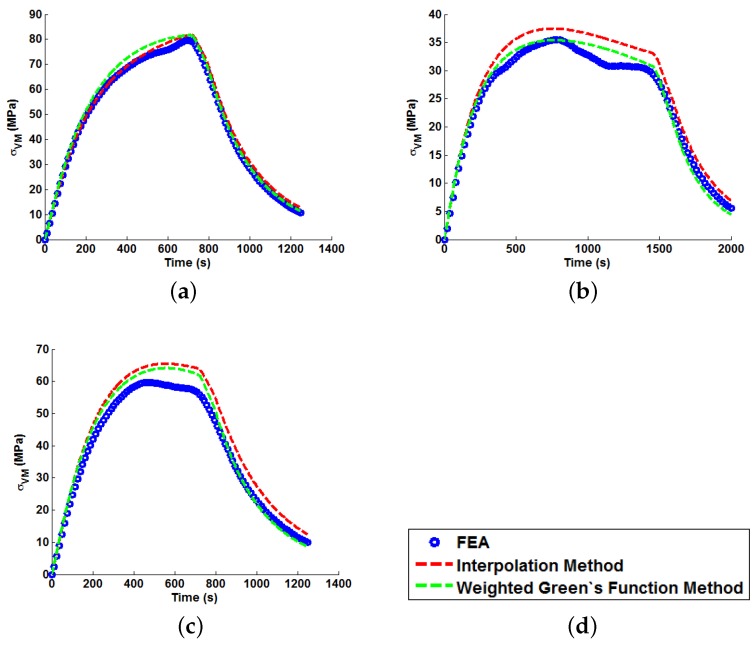
Predictions of thermal stress responses determined using the interpolation and weighted Green’s functions methods for: (**a**) ramp up Profile D; (**b**) ramp up Profile E; and (**c**) ramp up Profile F. Sub figure (**d**) shows the legend used in the plots.

**Figure 13:**
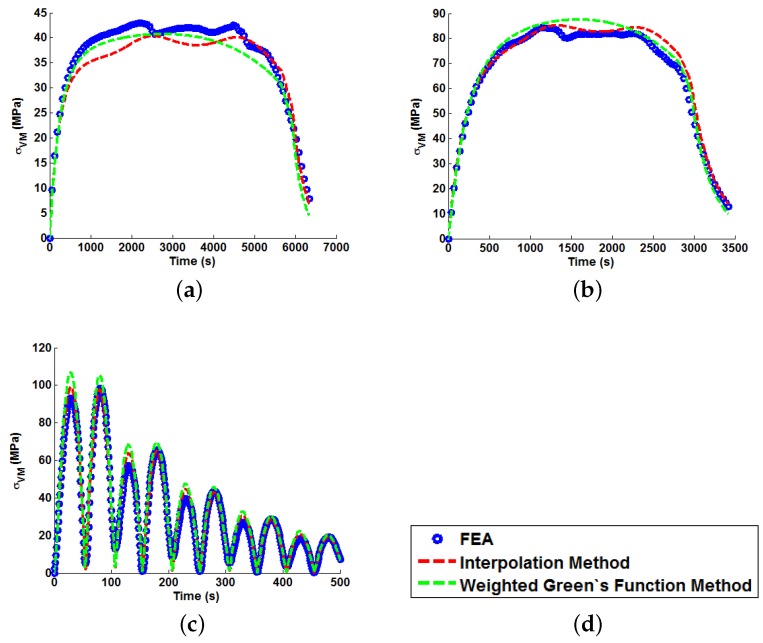
Predictions of thermal stress responses determined using the interpolation and weighted Green’s functions methods for: (**a**) ramp up Profile G; (**b**) ramp up Profile H; and (**c**) oscillating Profile I. Sub figure (**d**) shows the legend used in the plots.

In order to illustrate the importance of considering temperature dependency in the Green’s function approach, unit Green’s functions have been used individually to predict each of the nine temperature profiles A–I (thus highlighting the potential degree of stress over-/under-estimation). Plots of the true (FEA) and predicted thermal stresses may be seen in Figure 8, Figure 9 and Figure 10. Similarly, predictions of the thermal stress histories have been made using the temperature dependency techniques discussed (the weighting function and the proposed interpolation technique, shown in Figure 11, Figure 12 and Figure 13). In order to quantify the relative qualities of fit, coefficients of determination ($R^{2}$ [23]) and peak absolute differences between predicted and true stresses ($\Delta \sigma_{VM}$) have been determined for each method. These are summarised in Table 6 and Table 7, respectively.

**Table 6:** A summary of coefficients of determination ($R^{2}$) for the test temperatures profiles, fitted using the three Green’s function implementation methods.

Method	A	B	C	D	E	F	G	H	I
249 ℃–250 ℃	0.930	0.976	0.966	0.979	0.763	0.759	0.861	0.947	0.827
299 ℃–300 ℃	0.983	0.988	0.995	0.995	0.508	0.540	0.842	0.897	0.785
349 ℃–350 ℃	0.949	0.958	0.983	0.986	0.336	0.397	0.753	0.825	0.757
399 ℃–400 ℃	0.703	0.785	0.885	0.918	-0.185	-0.013	0.320	0.525	0.723
449 ℃–450 ℃	0.911	0.932	0.974	0.984	0.235	0.357	0.660	0.761	0.905
499 ℃–500 ℃	0.936	0.950	0.983	0.990	0.307	0.427	0.704	0.793	0.944
549 ℃–550 ℃	0.826	0.876	0.944	0.966	0.078	0.273	0.495	0.653	0.969
599 ℃–600 ℃	0.955	0.958	0.935	0.929	0.735	0.802	0.866	0.909	0.918
649 ℃–650 ℃	0.179	0.452	0.470	0.549	0.790	0.837	0.115	0.471	0.582
Interpolation Method	0.968	0.990	0.991	0.992	0.918	0.921	0.916	0.976	0.983
Weighting Method	0.986	0.967	0.989	0.990	0.979	0.967	0.940	0.976	0.955

**Table 7:** A summary of peak differences between true (FEA) and predicted stress values ($\Delta \sigma_{VM}$) for the test temperatures profiles, fitted using the three Green’s function implementation methods.

Method	A	B	C	D	E	F	G	H	I
249 ℃–250 ℃	5.09	6.65	3.95	5.90	10.49	19.36	8.94	16.50	31.59
299 ℃–300 ℃	2.63	5.37	1.68	2.40	13.49	24.79	11.97	22.20	35.63
349 ℃–350 ℃	3.54	8.47	3.07	5.02	15.02	27.56	13.52	25.10	37.95
399 ℃–400 ℃	6.73	15.92	6.74	11.06	18.68	34.02	17.26	32.22	41.15
449 ℃–450 ℃	4.14	10.61	4.03	5.40	15.98	28.36	14.70	27.80	26.72
499 ℃–500 ℃	3.68	9.64	3.52	4.60	15.45	27.07	14.25	27.12	21.80
549 ℃–550 ℃	5.59	13.37	5.29	7.63	17.22	29.56	16.19	31.06	17.26
599 ℃–600 ℃	3.86	8.19	5.28	9.94	10.69	16.58	10.34	19.86	24.43
649 ℃–650 ℃	11.39	21.86	12.04	23.09	8.01	13.15	10.94	22.38	46.83
Interpolation Method	2.76	6.14	2.53	3.69	4.72	7.85	4.34	9.36	9.75
Weighting Method	3.51	8.69	2.59	4.43	2.92	5.90	5.52	7.38	13.78

## 6. Discussion and Conclusions

**Figure 14:**
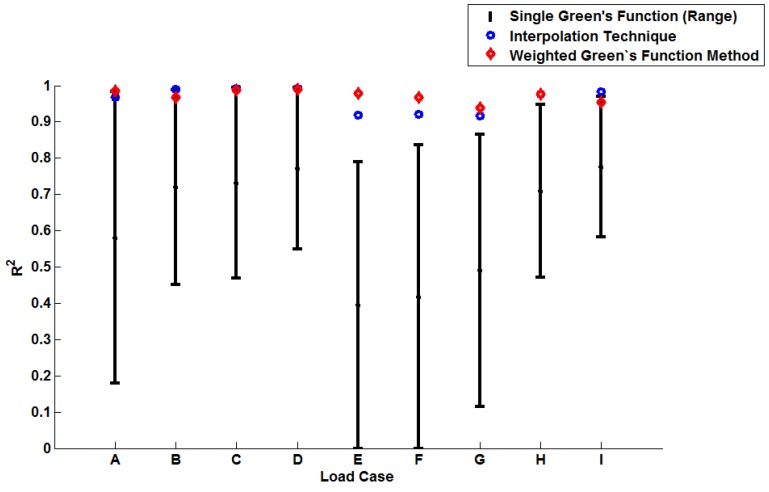
A plot to show the relative performance of the various implementation methods (using $R^{2}$).

**Figure 15:**
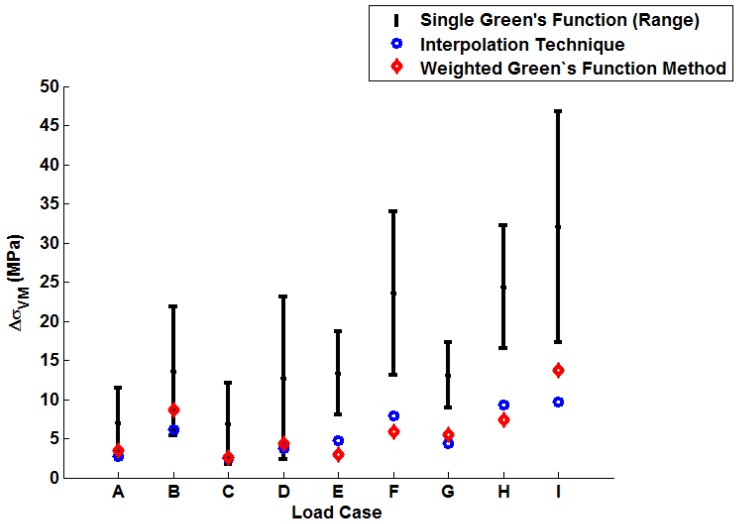
A plot to show the relative performance of the various implementation methods (using $\Delta \sigma_{VM}$).

The coefficient of determination ($R^{2}$) provides a statistical measure to describe the amount of variance accounted for in a model when predicting some “true” data (taking limiting values of zero if no variance is accounted for and one if all variance is accounted for [23]). The values determined for $R^{2}$ shown in Table 6 are plotted in Figure 14 (note the range of values determined when using individual Green’s functions is presented). Similarly, a plot of the peak instantaneous stress differences ($\Delta \sigma_{VM}$; see Table 7) is given in Figure 15. This quantity is of interest as, when attempting to quantify the threat of thermal fatigue using a reference elastic solution, stress ranges experienced in a component are one of the most fundamental ways to characterise a particular loading scenario.

In all cases, solutions where temperature dependency was considered in the Green’s function method showed an improvement in fitting quality. All results were at least in the 98th percentile of the range predicted when using individual Green’s functions (for the majority of cases, $R^{2}$ and $\Delta \sigma_{VM}$ values were better than even the optimum individual Green’s function solution). Despite the small number of load cases considered, some general comments can be made. $R^{2}$ values are comparable for most cases, and in almost all cases, the interpolation technique resulted in lower $\Delta \sigma_{VM}$ values than the weighting function technique (suggesting small stress over-/under-estimations). Anomalies to these observations are found in load Cases E and F, where the weighting function method appears to give superior results, represented by an approximate 2 MPa reduction in stress differences. It is also noted from Figure 11, Figure 12 and Figure 13 that load cases with large temperature increments (Cases A, B, G and H) do not result in smooth stress development curves that monotonically increase to a maximum value and then decay (*i.e.*, there are “ripples” in the thermal stress histories; see Figure 11b in particular). These features are not seen in more modest temperature step load cases.

**Figure 16:**
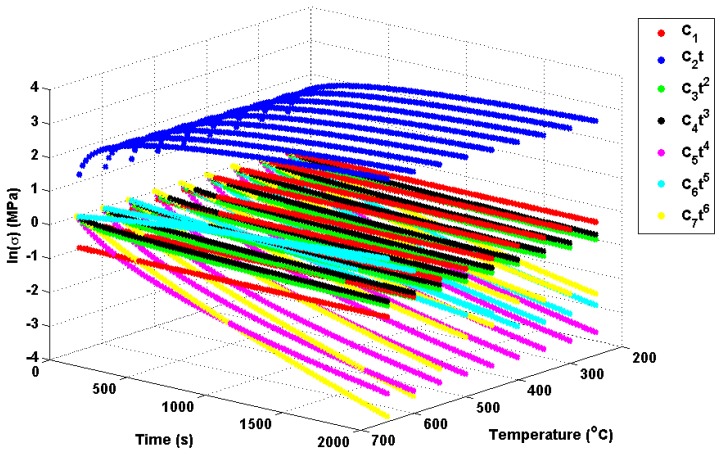
Decomposed Green’s functions, showing the relative importance of the exponential components.

Potential explanations for these behaviours can be found from analysis of the individual Green’s function components themselves and, in particular, the relative effects of the seven exponential components. These components are plotted in Figure 16 using the Green’s function coefficients given in Table 4. Of interest for the present work are the fifth and sixth order components (the $C_{6}$ and $C_{7}$ terms, respectively; most other components vary little over the given temperature range), which can be seen to (in part) control stress decay in the unit Green’s functions. Furthermore, there is a shift at around 450 ℃, with the sixth order component becoming more dominant in the decay characteristics and the fifth order component becoming positive (leading to a small contribution in stress development). Over large temperature ranges, multiple Green’s functions may be used with a wide range of decay characteristics. The “ripple” features discussed previously are therefore due to the complex (time dependent) interaction of these decay functions. While varying decay characteristics can be accounted for in the proposed interpolation method, the time-independent weight function assumes a scaling dependent only on temperature (hence, the superior fit observed for the interpolation method in Cases A, B, G and H). Cases C–F used temperature ranges between 450 and 650 ℃, where the relationship between components in Green’s function is reasonably linear (with temperature) and can therefore be accounted for using the weighting function. As the weighting function coefficients are estimated by an optimisation procedure using all unit responses, this linear relationship can be approximated, and local errors in the (particularly in the 450 ℃ and 650 ℃ profiles) can be minimised.

The proposed interpolation method has been shown to be adept at predicting thermal stress histories in thick-walled components. Over large temperature ranges (>150 ℃), where material properties may vary significantly, the interpolation procedure has out performed the weighting method. For more modest temperature ranges, the two methods are generally comparable. In addition to the increased generality that the interpolation procedure offers, it is worth highlighting important practical advantages. The order of the weighting function polynomial given in Equation (12) is ultimately dependent on the number of reference solutions generated. In the present work, nine unit temperature steps were applied to FEA models and used in the temperature dependency methods. Fewer reference solutions may be used in practice, however, due to practical limitations, potentially limiting the applicability of the weighting function. While a greater number of reference solutions is beneficial to the interpolation procedure, even a small number can be used to understand core variations in stress profiles with representative temperature. Some aspects of the second order effects may therefore also be captured. Future work will look to quantify the effect the number of available reference solutions has on the performance of the temperature dependency methods.

In conclusion, the present work has highlighted the importance of including temperature dependency in the Green’s function method in order to better estimate transient thermal stresses for realistic bulk steam temperature increments in thick-walled components. The proposed interpolation technique provides a general procedure to incorporate temperature dependency in Green’s function analysis. There is some suggestion from the results that the relative importance of each Green’s function component varies with temperature and leads to complex interactions between thermal stress development and decay terms. Over larger temperature ranges, this has been seen to have an effect; however, for small temperature ranges, the weighting function method proposed by Koo *et al*. appears to be satisfactory [17]. Future work will therefore focus on increasing the number of load cases considered in order to verify the suggested phenomenon and in accounting for spatial variations in material properties and chosen analysis points.
